# INFOMATAS multi-center systematic review and meta-analysis individual patient data of dynamic cerebral autoregulation in ischemic stroke

**DOI:** 10.1177/1747493020907003

**Published:** 2020-02-24

**Authors:** L Beishon, JS Minhas, R Nogueira, P Castro, C Budgeon, M Aries, S Payne, TG Robinson, RB Panerai

**Affiliations:** 1 CHIASM Group, Department of Cardiovascular Sciences, University of Leicester, Leicester, UK; 2 NIHR Leicester Biomedical Research Centre, British Heart Foundation Cardiovascular Research Centre, Glenfield Hospital, Leicester, UK; 3 Neurology Department, School of Medicine, Hospital das Clinicas, University of São Paulo, São Paulo, Post Brazil; 4 Stroke Unit and Department of Neurology, Centro Hospitalar Universitário São João, Porto, Portugal; 5 Department of Intensive Care, University Maastricht, Maastricht University Medical Center, Maastricht, Netherlands; 6 Institute of Biomedical Engineering, Department of Engineering Science, University of Oxford, Oxford, UK

**Keywords:** Cerebral autoregulation, ischemic stroke, cerebral hemodynamics, blood pressure, meta-analysis, autoregulation index

## Abstract

**Rationale:**

Disturbances in dynamic cerebral autoregulation after ischemic stroke may have important implications for prognosis. Recent meta-analyses have been hampered by heterogeneity and small samples.

**Aim and/or hypothesis:**

The aim of study is to undertake an individual patient data meta-analysis (IPD-MA) of dynamic cerebral autoregulation changes post-ischemic stroke and to determine a predictive model for outcome in ischemic stroke using information combined from dynamic cerebral autoregulation, clinical history, and neuroimaging.

**Sample size estimates:**

To detect a change of 2% between categories in modified Rankin scale requires a sample size of ∼1500 patients with moderate to severe stroke, and a change of 1 in autoregulation index requires a sample size of 45 healthy individuals (powered at 80%, α = 0.05). Pooled estimates of mean and standard deviation derived from this study will be used to inform sample size calculations for adequately powered future dynamic cerebral autoregulation studies in ischemic stroke.

**Methods and design:**

This is an IPD-MA as part of an international, multi-center collaboration (INFOMATAS) with three phases. Firstly, univariate analyses will be constructed for primary (modified Rankin scale) and secondary outcomes, with key co-variates and dynamic cerebral autoregulation parameters. Participants clustering from within studies will be accounted for with random effects. Secondly, dynamic cerebral autoregulation variables will be validated for diagnostic and prognostic accuracy in ischemic stroke using summary receiver operating characteristic curve analysis. Finally, the prognostic accuracy will be determined for four different models combining clinical history, neuroimaging, and dynamic cerebral autoregulation parameters.

**Study outcome(s):**

The outcomes for this study are to determine the relationship between clinical outcome, dynamic cerebral autoregulation changes, and baseline patient demographics, to determine the diagnostic and prognostic accuracy of dynamic cerebral autoregulation parameters, and to develop a prognostic model using dynamic cerebral autoregulation in ischemic stroke.

**Discussion:**

This is the first international collaboration to use IPD-MA to determine prognostic models of dynamic cerebral autoregulation for patients with ischemic stroke.

## Background

**Figure 1.**
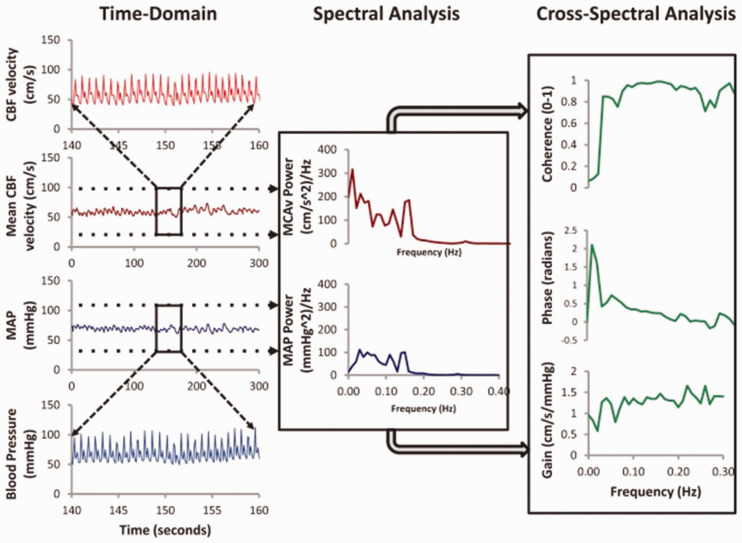
TFA metrics of gain, phase, and coherence.^20,2^ MAP: mean arterial pressure; MCAv: cerebral blood flow-velocity in the middle cerebral artery; CBF: cerebral blood flow.

Dynamic cerebral autoregulation (dCA) is a key homeostatic mechanism to maintain a constant cerebral perfusion despite systemic fluctuations in blood pressure (BP) and CO_2_.^1^ dCA can be measured non-invasively by transcranial Doppler ultrasonography (TCD), most commonly using transfer function analysis (TFA) of spontaneous fluctuations in BP.^1,2^ A number of excellent reviews have been published on TFA,^2,3^ but in brief, TFA uses Fourier decomposition to measure three main properties of autoregulation: gain, phase, and coherence.^2^ In addition, the autoregulation index (ARI) can also be calculated using the gain and phase frequency responses. Gain describes the frequency-dependent ratios in amplitude of the input (BP) compared to the output (cerebral blood flow velocity, CBFv), where higher gain represents less efficient autoregulation.^2^ Phase shift describes the recovery of changes in CBFv relative to those in ABP, where high phase shift represents more efficient autoregulation.^2^ Coherence expresses the fraction of output (CBFv) power that can be linearly explained by the corresponding input (BP) power at each frequency. Values of coherence approaching 1.0 result from signals with very high signal-to-noise ratio (SNR) and a strong univariate input–output linear relationship. The 95% confidence limit of the distribution of coherence is normally used to reject estimates of gain and phase where SNR is low, the relationship is non-linear, or there are multiple inputs affecting the CBFv output.^2^ Figure 1 summarises the derivation of the key TFA metrics of gain, phase, and coherence.

Systemic and cerebral hemodynamic perturbations in the acute/sub-acute phase of ischemic stroke (IS) may affect clinical outcomes,^3,4^ with possible correlations with stroke severity.^5^ Several single-center TCD studies primarily investigating IS sub-types have increased our understanding of dCA during the acute and chronic phases.^3^ Unfortunately, prior attempts at data synthesis have failed due to a lack of methodological homogeneity in TCD study design and analysis.^3^ However, large normative datasets,^6^ consensus guidelines,^2^ and multi-center studies to improve reproducibility of CA estimates^7^ are examples of efforts designed to minimize heterogeneity and increase statistical power. There exist convergent findings demonstrating impairment of CA up to two weeks post IS. Importantly, these findings are demonstrated irrespective of geographical location^8^ and the presence of hypocapnia.^4^ Increased emphasis is being placed on “dynamic” studies of blood flow during the acute stroke period in an effort to determine the impact of autoregulation-guided management strategies on clinical outcome. Concern has been raised about “intensive” strategies to lower BP, particularly in the presence of presumed impaired autoregulation, as there is the potential for ischemic harm through cerebral hypoperfusion. The 2nd CARNet Bootstrap Project has pooled data from five different centers,^9^ but at present, there has been no pooled individual patient analyses with dCA data of IS patients.

Individual patient data meta-analysis (IPDMA) contrasts with traditional methods for meta-analysis by aggregating raw data at the individual patient level rather than combining study-level data.^10,11^ IPDMA is increasingly used in areas previously hampered by significant heterogeneity at study-level, thus improving standardization of analysis across studies, reducing heterogeneity, and improving reliability of pooled estimates.^10,11^ IPDMA can be considered as a one- or two-stage approach; the former uses statistical methods to construct multivariate models, where patients are clustered by study origin using mixed-effects modelling.^10^ In the two-stage approach, data are re-analyzed at the individual level and traditional methods are employed to aggregate the data at study level.^10^ There is an increasing trend in the literature towards the use of one-stage IPD analysis over the traditional two-stage method.^12,13^ The one-stage approach has been demonstrated recently to out-perform the two-stage approach, particularly when investigating interaction effects. In the context of dCA measurements in IS, many of the primary studies are case-control, cross-sectional, or cohort in design, with small patient numbers.^3^ Thus, adjustment for confounders in primary studies is frequently hampered by small sample sizes. A one-stage approach would facilitate an adjusted analysis of the role of dCA in IS and provide important information on which baseline factors are associated with better or worse dCA in IS, and how this relates to prognosis and clinical outcomes.

This analysis has been separated into three distinct phases with the following aims:

1. To explore the scope, severity, and temporal changes of dCA impairments in IS, and the relationship with baseline demographics, and neuroimaging and clinical outcome variables
2. To identify the diagnostic test accuracy (DTA) for measures of dCA impairment in distinguishing IS from non-IS and in predicting outcome
3. To develop a risk prediction model for outcome in IS by combining clinical, neuroimaging, and dCA information.

## Methods

This protocol has been developed in line with reporting guidelines for IPD analyses.^14^

### Inclusion criteria

1. Adults aged >18 years
2. Diagnosis of IS (all sub-types)
3. Cerebrovascular parameters available, including indices of dCA (up to 12 months post-symptom onset).

### Exclusion criteria

1. Center declines to participate
2. Significant missing data that will compromise study validity determined by consensus agreement of the INFOMATAS group
3. Low-quality studies as per criteria below.

### Identification of participating centers

Participating centers’ contributing data analysis will be identified through the Cerebral Autoregulation Network, from recent systematic reviews, and through systematic searching of the literature (search strategy in Supplementary Information I).

### Creation of IPD file and confidentiality

All individual patient data will be anonymized prior to sharing between centers, and no patient identifiable data will be included. Ethical approval for this study was not sought as no new patient data are being collected, and analyses are similar to those conducted in the original individual trials.

### Data exchange

A data use agreement will be in place prior to data exchange. A list of the selected variables which will be shared between centers is shown in Supplementary Information II. A standardized data dictionary will be used by all centers to ensure variables are collected and coded in a consistent manner between centers. The receiving center will amalgamate the data independently of the analyzing center.

### Data quality assurance

Data will be checked at the contributing center level for accuracy, completion, and integrity. The nomenclature of the files will be standardized between centers prior to analysis. Any data queries or missing data will be resolved through contact with the trial investigators.

### Pooled IPD analysis sample

Studies included in the final analysis will be summarized, in terms of inclusion and exclusion criteria and baseline characteristics.

### Primary study quality and risk of bias assessment

The primary studies included in the final analysis will be appraised for quality using CONSORT^15^ (randomized), STROBE^16^ (observational), QUADAS-2 (DTA) studies,^17^ and against the criteria in the CARNet white paper for studies of dCA.^2^ Two centers will independently appraise studies and a third center will mediate disagreements in quality assessment. Risk of bias will be summarized in table and charts.

### Baseline dCA variables

In addition to standard parameters used to describe cerebral hemodynamics (mean CBFv, mean arterial pressure, end-tidal CO_2_ (EtCO_2_), heart rate), the metrics adopted to assess dCA will be those obtained from TFA of recordings at rest (coherence, gain, phase, both at very low frequency and low frequency), as well as the ARI (from TFA, thigh cuff or sit-to-stand). The methods used by primary studies to generate these measurements will be summarized.

### Baseline co-variates

Co-variates relating to participant characteristics, interventions, and neuroimaging findings recorded at baseline will be included in the analyses. A full list of all co-variates and descriptors can be seen in Supplementary Information II.

### Outcome variables

The temporal changes in dCA parameters (gain, phase, ARI) will be investigated relative to baseline measurements from the acute phase, where data are available, up to 12 months post-IS. Where available, these differences will be compared to control population data. We will identify which baseline parameters are predictive of poorer dCA in the longer term, and whether this relates to clinical outcome (modified Rankin scale (mRS)) using uni- and multivariable analyses as described below.

The primary time point for analysis of primary and secondary clinical and dCA outcomes will be three months, but we anticipate that studies will have reported outcomes at different time points and will thus be grouped into time points from symptom onset, where the pathophysiological changes are similar to facilitate analyses: within the first 24 h, 24–72 h, 4–7 days, and 3, 6 and 12 months post-event. If possible, the first 24 h will be further sub-divided into 0–6 h and 7–24 h.

The primary outcome for the analysis will be mRS. Secondary clinical outcome measures include: mortality, mRS (dead or dependent (3–6)/independent (0–2)), National Institute for Health stroke scale (NIHSS), Glasgow Coma Scale (GCS), Barthel index, symptomatic hemorrhagic transformation, and infarct size/volume will be collected from the last radiological imaging (MRI or CT) undertaken during hospital stay. A full list of all outcomes and descriptors can be seen in Supplementary Information II.

### Missing data

The investigators anticipate that the majority of datasets will be complete; however, datasets with missing data will be considered for analysis if they meet the quality criteria and will be determined by consensus agreement of the INFOMATAS group.

### IPD analysis

#### Phase 1: Univariable analysis

The primary outcome (mRS) and all other binary or ordinal outcomes will be analyzed using Generalized Estimating equations. All continuous outcomes will be analyzed using Generalized Linear Mixed Models. All models will consider the study from which the patient originated as a random effect. All models will consider the co-variates outlined above. Model estimates, standard errors, odds ratios, and 95% confidence intervals will be presented where appropriate. Results will be considered statistically significant where p < 0.05.

#### Phase 2: DTA

In the first instance, data will be analyzed at study level and extracted into binary two-by-two tables (binary test results cross-classified with the binary reference standard) to calculate sensitivities and specificities with 95% confidence intervals for each parameter of dCA. For each study, estimates of sensitivity and specificity will be plotted graphically in forest plots and receiver operating characteristic (ROC) curves (RevMan 5). Where there are more than three studies available at the same test threshold and parameter, summary analyses will be performed using a bivariate random effects analysis, to calculate pooled estimates of sensitivity, specificity, positive and negative predictive values, positive and negative likelihood ratios, with 95% confidence intervals. The test thresholds will be identified through systematic review and through consensus discussion with the INFOMATAS group.

Summary analysis will be performed using MetaDTA.^18^

#### Phase 3: Multivariable modelling and DTA analysis

Using the univariable analysis from phase 1, four models will be constructed to investigate the test properties for each of the dCA parameters when prognostic patient factors are accounted for. The four models will be constructed as follows:

Model 1: clinical history alone
Model 2: clinical history + dCA parameters
Model 3: clinical history + neuroimaging
Model 4: clinical history + neuroimaging + dCA parameters

Each of the above models for different dCA parameters will be represented graphically in a ROC curve, and the accuracy of each model will then be compared from the ROC analysis.

### Heterogeneity analyses

Pre-specified heterogeneity, sub-group and sensitivity analyses have been presented in Supplementary Information III.

#### Sample size

Firstly, to detect a change of 1 in ARI, a sample of at least 45 healthy individuals is required (powered at 80%, α = 0.05). We anticipate a loss to follow-up of approximately 10% of the cohort and thus require a sample of at least 65 stroke patients. Secondly, to detect a change of 2% between categories in mRS, a sample size of approximately 1500 patients is required in moderate to severe stroke (powered at 80%, α = 0.05).^19^ To date, no study has calculated the required sample size to detect clinically significant change in dCA parameters in IS, and individual studies have thus far been small; therefore, accurate mean and standard deviation values are not known. We plan to use the pooled mean and standard deviation from this IPDMA to undertake sample size calculations for future studies to facilitate adequately powered studies using dCA parameters as outcome measures.

## Supplemental Material

WSO907003 Supplemental material - Supplemental material for INFOMATAS multi-center systematic review and meta-analysis individual patient data of dynamic cerebral autoregulation in ischemic stroke Click here for additional data file. Supplemental material, WSO907003 Supplemental material for INFOMATAS multi-center systematic review and meta-analysis individual patient data of dynamic cerebral autoregulation in ischemic stroke by L Beishon, JS Minhas, R Nogueira, P Castro, C Budgeon, M Aries, S Payne, TG Robinson and RB Panerai in International Journal of Stroke

## Collaborators

Corina Puppo, Li Xiong, Nathalie Nasr, Shieak Tzeng, Andrew Robertson, Angela Salinet, Nils Petersen, Jia Liu, Zhen-Ni Guo, Yi Yang, Yi Yang, Zhen-Ni Guo, Jia Liu, Erik Gommer, Joseph Miller, Nai-Fang Chi, Martin Mueller, Caroline Rickards.
