# Supplementary material for: INFOMATAS multi-center systematic review and meta-analysis individual patient data of dynamic cerebral autoregulation in ischemic stroke
Source: Int J Stroke. 2020 Feb 24;15(7):807–12. doi: 10.1177/1747493020907003 (PMC7534203; doi:10.1177/1747493020907003)
Supplement: WSO907003 Supplemental material - Supplemental material for INFOMATAS multi-center systematic review and meta-analysis individual patient data of dynamic cerebral autoregulation in ischemic stroke [file WSO907003_Supplemental_material.pdf]

Supplementary Information I – Search Strategy

The search strategy will be piloted initially, and if insufficient articles are returned will be revised and re-piloted. The search will be limited to studies in humans, but not by language. We will search the following databases: Medline, Embase, Web of Science, Cochrane database, PsychINFO. Reference lists of included articles will also be searched and we will use the Pubmed "related articles" feature to identify additional literature. Citation searching will be completed with tools such as Web of Science and Scopus. We will search grey literature databases and contact authors working in the field to identify any additional, unpublished data.

Population

Acute ischaemic stroke

Acute stroke

Stroke

AIS

Intervention

Cerebral blood flow*

dCA

Cerebral autoregulat*

Dynamic cerebral autoregulat*

Cerebral haemodynamic*

Autoregulatory index

ARI

Transfer function analysis

TFA

Phase

Gain

Coherence

Sit to stand

Squat stand

Thigh cuff

Outcome

Modified Rankin Scale

mRS

Mortality

Death

Dependent

National Institute of Stroke Severity Scale

NIHSS

Glasgow Coma Scale

GCS

Barthel

Infarct size

Infarct volume

Infarct extension

Infarct growth

Haemorrhagic transformation

Parenchymal haematoma

Cerebral oedema

Supplementary Information II – list of all pre-defined variables for which data will be collected

Demographic variables:

Age

Sex

Ethnicity

Time to randomisation

Time to thrombolysis/thrombectomy

Glasgow Coma Scale (GCS)

National Institute of Health Stroke Severity Scale (NIHSS)

Modified Rankin Scale (mRS) (baseline and pre-morbid)

Barthel index

Medical co-morbidities: diabetes mellitus, hypertension, previous stroke, atrial fibrillation, carotid artery disease (present/absent at >70% (NASCET criteria (1)), ischaemic heart disease, heart failure (with and without reduced ejection fraction), smoker, antihypertensive and statin therapy, frailty (as determined by a clinical frailty score, e.g. Clinical Frailty Scale (2)).

Stroke characteristics:

Affected hemisphere

Sub-type (large vessel occlusion (LVO)/non-LVO),

CT angiography (ICA, T, M1, M2 or M3)

Haemorrhagic transformation (European Cooperative Acute Stroke Study (ECASS II) classification (3))

Oedema

Physiological parameters:

Heart rate, blood pressure

End-tidal CO_2 ($EtCO_2$)

Outcome variables:

mRS

Phase

Gain

ARI

Mortality

mRS (Dead or dependent/independent)

NIHSS

GCS

Barthel

Infarct size/volume

Infarct extension (infarct growth measured as the difference in in infarct volume between baseline and follow-up (4))

Predicted or actual infarct size/volume (Alberta Stroke Program early CT score (ASPECTS) at baseline (5))

Haemorrhagic transformation (ECASS II criteria subtypes: HT1, HT2, and parenchymal haematoma (PH) type 1 and 2 (3))

Cerebral oedema

Supplementary Information III – Pre-specified heterogeneity and sensitivity analyses

Heterogeneity analyses

Heterogeneity will be investigated using the I^2 index (6), where I^2 <25% corresponds to low, 50% moderate, and >75% high heterogeneity, and is summarised in the following equation:

$$I^2 = [Q - df/Q] \times 100$$

Where: *Q* is Chi-square statistic, df is degrees of freedom.

Heterogeneity will be considered significant where $p<0.05$. The traditional method of I^2 was originally developed for a two-stage analysis, but has recently been adapted for one-stage IPD analyses (7).

In addition to quantitative evaluation of heterogeneity, the following areas will be investigated as part of a pre-specified analysis:

Case mix

It is anticipated there will be significant differences between studies in terms of: the age of participants, their ethnicity, study location, severity and type of stroke, and co-morbidities. The relationship between dCA parameters and clinical outcome are likely to vary significantly between these factors, and will be investigated through pre-specified sub-group analyses outlined below.

Stroke diagnosis and assessment

There is likely to be significant heterogeneity between centres in the methods and classifications used to diagnose and sub-type AIS. The quality of stroke assessment and diagnosis will be assessed using the quality assessment tools (QUADAS-2, STROBE, and CONSORT).

dCA assessment

There is likely to be significant differences between centres in the protocols used to collect and analyse the dCA parameters. Data will be collected on the protocols and procedures used by individual centres for the collection and analysis of dCA parameters.

Pre-specified sub-group analyses

Where sufficient data are present, pre-specified analyses will include the following:

1) The relationship between key risk factors, dCA parameters and outcome in AIS including
2) The relationship between outcome from intervention (thrombolysis and thrombectomy) and dCA parameters in AIS
3) The relationship between neuroimaging outcomes and dCA parameters in AIS
4) The relationship between outcome and dCA parameters at varying time points
5) DTA test properties will be investigated within high risk sub-groups

Sensitivity analysis

Sensitivity analyses will be performed to identify the effect of any studies classified at high risk of bias on summary estimates. Initially, all studies will be included in analyses, and those identified to be at high risk of analysis will be removed, and the summary statistics re-calculated.

References

1. Barnett HJM, Taylor DW, Haynes RB, et al. Beneficial effect of carotid endarterectomy in symptomatic patients with high-grade carotid stenosis. N Engl J Med 1991;325(7):445-53.
2. Rockwood K, Song X, MacKnight C, et al. A global clinical measure of fitness and frailty in elderly people. Cmaj 2005;173(5):489-95.
3. Zhang J, Yang Y, Sun H, Xing Y. Hemorrhagic transformation after cerebral infarction: current concepts and challenges. Annals of translational medicine 2014;2(8):81.
4. Harston GWJ, Carone D, Sheerin F, Jenkinson M, Kennedy J. Quantifying Infarct Growth and Secondary Injury Volumes: Comparing Multimodal Image Registration Measures. Stroke 2018;49(7):1647-55.
5. Pexman JH, Barber PA, Hill MD, et al. Use of the Alberta Stroke Program Early CT Score (ASPECTS) for assessing CT scans in patients with acute stroke. AJNR Am J Neuroradiol 2001;22(8):1534-42.
6. Higgins J, Green S. Cochrane Handbook for Systematic Reviews of Interventions. 5.1.0 ed: The Cochrane Collaboration, 2011.
7. Chen B, Benedetti A. Quantifying heterogeneity in individual participant data meta-analysis with binary outcomes. Systematic Reviews 2017;6(1):243.
